# Species-specific responses of young deciduous and coniferous trees to simulated particulate matter

**DOI:** 10.3389/fpls.2025.1622995

**Published:** 2025-10-08

**Authors:** Iveta Varnagirytė-Kabašinskienė, Valentinas Černiauskas

**Affiliations:** Lithuanian Research Centre for Agriculture and Forestry, Department of Silviculture and Ecology, Institute of Forestry, Kaunas District, Lithuania

**Keywords:** air pollution, PM simulation, tree seedlings, stem growth, physiological stress

## Abstract

**Introduction:**

Particulate matter (PM) is a significant air pollutant associated with severe health and environmental issues. Although urban trees help filter PM through their leaves and surfaces, PM pollution disrupts their structure and function at various levels, affecting photosynthesis, blocking stomata, and inducing oxidative damage.

**Methods:**

This study evaluated the growth, biomass, and physiological responses of five tree species - silver birch (*Betula pendula*), small-leaved lime (*Tilia cordata*), Norway maple (*Acer platanoides*), Scots pine (*Pinus sylvestris*), and Norway spruce (*Picea abies*) - to artificial PM exposure. One- to two-year-old seedlings were divided into control and PM-treated groups.

**Results and Discussion:**

Norway maple and small-leaved lime were the most resilient, maintaining growth and activating stress defences. Silver birch showed moderate tolerance, with biochemical compensation despite growth suppression. Norway spruce experienced a moderate decline in physiological balance and growth. Scots pine was the most sensitive, displaying reduced growth and heightened oxidative stress. The study highlighted the importance of species selection for urban planting. Due to their PM tolerance, Norway maple and small-leaved lime appear to be best suited for polluted environments. Silver birch and Norway spruce may be suitable for moderately polluted areas, while Scots pine is less ideal for high-pollution urban settings. These findings support the concept of environmental hormesis, where low-dose stressors elicit adaptive responses in tolerant species. However, the observed species-specific responses and the broader applicability of the results may be constrained by several factors, including the use of relatively young seedlings, the limited duration of exposure, and the specific method of simulating PM pollution.

## Introduction

Particulate matter (PM) is a critical pollutant that induces environmental and health problems. Extremes have the worst consequences, threatening plant growth and productivity, causing long-term health effects, and premature deaths (Roy et al., 2024; WHO, 2025). PM significantly threatens plant growth and productivity by affecting their morphological, anatomical, and physiological functions (Rai, 2016; Roy et al., 2024). Conversely, trees and forests in urban areas serve as natural biofilters, capturing PM through physical and physiological processes (Manes et al., 2016; Tiwari et al., 2019; Kim et al., 2022). PM has a broad distribution regarding size and diverse components, classified as organic and inorganic pollutants, as summarized by Bell et al. (2011). Due to its complex chemical composition, the toxicity of particulate matter (PM) varies depending on its emission sources, such as vehicle traffic, industrial processes, and biomass burning, as well as environmental conditions (Allouche et al., 2024). Previous studies highlighted that the presence of trees can reduce PM_2.5_ concentrations within urban forests (Zhu et al., 2019; Corada et al., 2021; Kim et al., 2022). Key characteristics influencing PM capture include leaf micromorphology, surface roughness, and wax composition (Leonard et al., 2016; Chen et al., 2024). Therefore, the PM retention ability varies among species, and careful selection of plant species for urban air quality management is necessary (Nowak et al., 2014; Kim et al., 2022; Barwise and Kumar, 2020). The importance of urban trees as an effective tool for mitigating air pollution is widely discussed (Sæbø et al., 2012; Popek et al., 2017; Łukowski et al., 2020), but the effectiveness of such mitigation efforts is often limited by the physiological stress caused by PM on the trees (Roy et al., 2024). PM blocks stomata, reducing gas exchange, photosynthetic rates, and plant growth by limiting photosynthetically active radiation (Yu et al., 2018; Przybysz et al., 2014; Rai, 2016; Li et al., 2023; Roy et al., 2024). This disruption in stomatal function and metabolism results in pigment loss, antioxidant enzyme changes, and membrane damage in trees (Chaudhary and Rathore, 2018). Tree seedlings exposed to PM frequently activate stress-response mechanisms, producing antioxidants to counteract oxidative damage, though prolonged exposure can inhibit growth (Sæbø et al., 2012).

For example, species such as *Quercus robur* L. and *Betula pendula* Roth effectively trap PM due to their dense foliage and surface properties, while conifers such as *Pinus sylvestris* show better PM retention due to their needles, despite their susceptibility to pollution stress (Sæbø et al., 2012; Mo et al., 2015). Broad-leaved species, such as *Platanus × acerifolia* (Aiton) Willd., also respond to high PM retention due to their large leaf surface area and wax properties (Chen et al., 2024).

Understanding the interactions between PM and tree seedlings is essential for optimizing species selection for greenery planning in urban areas, particularly for air quality improvement initiatives (Barwise and Kumar, 2020). Therefore, we aimed to evaluate the growth and biochemical responses of seedlings of three deciduous tree species, such as silver birch (*Betula pendula* Roth), small-leaved lime (*Tilia cordata* Mill.), and Norway maple (*Acer platanoides* L.), and two coniferous tree species, such as Scots pine (*Pinus sylvestris* L.) and Norway spruce (*Picea abies* (L.) H.Karst.), to an artificial exposure to PM pollution.

## Materials and methods

A field experiment was designed to cover two vegetation seasons, from the beginning of 2023 till the end of 2024.

The average annual air temperature was 8.7°C (1.3°C higher than the standard climate normal - SCN, calculated as the mean of the value during 1991–2020) in 2023 and 9.5°C (2.1°C higher than SCN) in 2024 (LHMT, 2024). The most significant deviations from the SCN occurred in August and September 2023, with August recording an average monthly air temperature of 19.5°C (+1.9°C above the SCN) and September averaging 16.5°C (+3.7°C above the SCN). The average annual precipitation was comparable to SCN (695 mm), with 719 mm (3% higher than SCN) in 2023 and 624 mm (10% lower than SCN) in 2024. The mean monthly temperatures and precipitation next to the SKN of the corresponding indicator in the Kaunas meteorological station are given in **Table 1**.

**Table 1:** Mean monthly temperature (°C) and precipitation (mm) in Kaunas, Lithuania (54.9°N, 23.91°E; 74 m a.s.l.): Observations for 2023–2024 and standard climate normals (SCN, 1991–2020) (LHMT, 2024).

Year	Months											
	Jan	Feb	Mar	Apr	May	Jun	Jul	Aug	Sep	Oct	Nov	Dec
Mean monthly temperature, °C												
2023	0.9	0.0	2.9	8.5	12.7	17.3	17.9	20.2	17.1	8.3	2.3	0.5
2024	-3.1	-2.6	1.4	7.8	13.0	16.3	18.8	18.0	13.2	7.7	2.6	-1.6
SCN^1^	-3.0	-2.4	1.2	7.6	13.0	16.3	18.6	17.8	12.9	7.2	2.6	-1.2
Mean monthly precipitation, mm												
2023	66.5	27.5	30.8	26.7	14.3	64.0	36.8	96.2	11.6	99.1	30.4	47.0
2024	47.8	37.7	37.5	34.4	56.4	66.1	84.6	79.3	49.7	58.8	45.4	43.5
SCN	48.0	38.0	38.0	38.0	53.0	65.0	88.0	77.0	51.0	61.0	47.0	47.0

^1^ Standard Climate Normals (1991–2020) from the Lithuanian Hydrometeorological Service (LHMT, 2024).

### Planting material and growth conditions

Seedlings of Scots pine, Norway spruce, silver birch, small-leaved lime, and Norway maple, which are native species in the hemiboreal forest zone and usually cultivated in urban areas, were selected for this study. One- to two-year-old tree seedlings were purchased from tree nurseries, which grow tree seedlings for reforestation, afforestation, and urban greening projects (**Table 2**).

**Table 2:** Characteristics of planting material.

Seedlings	Age, years	Nursery	Seed origin
Scots pine	1	Nemenčinės; State Forest Enterprise (Vilnius, Lithuania)	Eastern Lithuania
Norway spruce	1	Dubravos; State Forest Enterprise (Vilnius, Lithuania)	Central Lithuania
Silver birch	2	Strošiūnų; State Forest Enterprise (Vilnius, Lithuania)	Central Lithuania
Small-leaved lime	2	Kuršėnų; State Forest Enterprise (Vilnius, Lithuania)	Northern Lithuania
Norway maple	2	Kołaki–Wietrzychowo; Regional Directorate of State Forests (Białystok, Poland)	Northeastern Poland

For this study, plastic base-perforated pots were filled with commercially available nutrient-rich peat substrate (pH 5.5-6.5) (SuliFlor SF2, Sulinkiai, Lithuania). Seedlings were individually planted in pots in April 2022 and grown for one year before the experiment. They were grown in an open field without light or temperature control and irrigated as needed. After one year of growth, all tree seedlings were received NPK liquid fertilizer supplemented with Cu and Zn (Baltic Agro, Lithuania) at the recommended dosage.

### Treatments

In April 2023, the seedlings were divided into the particulate matter (PM) treatment group and the untreated group (control). Twenty visually healthy seedlings with standard growth characteristics were selected for each species from each PM and control group.

To simulate PM pollution, dry, solid and dusty material with a size of PM particles less than 10 µm (ISO 13322-1:2014) obtained from a heating boiler multicyclone in Girionys (Kaunas district, Lithuania) was used. The treatment material originated from forest biomass used for energy. Chemical content of the treatment material is available from Černiauskas et al. (2025). At the beginning of the experiment (May 2023), each PM-group seedling was first exposed to 0.4 g of PM. The dusty PM was manually applied evenly from above the seedlings during the daytime under similar weather conditions, avoiding rainy and windy days. The experiment spanned two vegetation seasons, with active PM treatments applied every 8 ± 2 days from May to August each season. Control-group seedlings were grown in similar conditions, ensuring they were placed sufficiently far from the PM particle dispersion zone. The experimental area was in a clean, pollution-free environment near a forest, eliminating potential urban air pollutants. This study design ensured that all seedlings were consistently and simultaneously exposed to urban PM pollution, compared to an environment with no elevated PM levels. Although this method does not fully represent the variability of PM deposition in real urban areas, it provides a simplified scenario with higher PM levels that seedlings contact when growing near roads and other pollution sources.

### Measurements

#### Seedling stem and biomass measurements

Seedling stem height (cm) was measured with a metric ruler, and diameter (mm) at the stem base was measured using digital callipers: at the beginning of the first vegetation season (before the first PM treatment, in April 2023), at the end of the first vegetation season (September 2023) and at the end of the second vegetation season (September 2024).

At the end of the experiment (September 2024), the aboveground biomass of each seedling from both the PM and control groups was measured for all seedlings. The biomass measurements were conducted by evaluating the dry mass of each seedling’s stem, branches, and foliage compartments separately, and the total biomass was calculated by summing the obtained masses per seedling. Then, mean values per PM and control groups for each tree species were calculated. Since the seedlings were relatively small, all sampled material was dried at 100–105°C until a constant mass and then weighed.

#### Biochemical analyses of seedlings

Biochemical analyses were conducted on foliage samples from both the PM and control group seedlings twice: at the end of the first and second vegetation seasons. Current-year/grown-for-one-season foliage (needles for coniferous and leaves for deciduous) was sampled on the same day (end of July 2023 and 2024). Samples were collected from nine randomly selected PM and Control group seedlings per tree species. The sampled foliage was intact, fully formed, and undamaged. Each sample was pooled from 15–20 needles and 5–10 leaves and stored at -20°C for up to 1 month for biochemical analyses (Malagoli et al., 2022; Čėsnienė et al., 2023). The standard and modified TPC methodology for biochemical analyses used in this study were described in detail previously by Beniušytė et al. (2023); Čėsnienė et al. (2023); Sirgedaitė-Šėžienė et al. (2024) and Černiauskas et al. (2025).

For extract preparation, a total of 0.1 g of foliage biomass was pulverized using “Precellys 24” (Bertin Technologies, France) tissue homogenizer at 1956 × g for 30 s and then poured over with 2 ml of 80% (v/v in water) ethanol. The samples were centrifuged for 30 min, 21,910 × g, +4°C, using a Hettich Universal 32R centrifuge (Andreas Hettich GmbH & Co. KG, Tuttlingen, Germany). The supernatant was used to measure the contents of photosynthetic pigments (Chl a and b, carotenoids), total polyphenol (TPC), total flavonoid (TFC), total soluble sugars (TSS), and malondialdehyde (MDA).

Chlorophyll a and b, and carotenoids were determined spectrophotometrically at 470 nm, 648 nm and 664 nm (using SpectroStar Nano microplate reader (BMG Labtech, Offenburg, Germany). Absorption was measured at 470 nm, 648 nm, and 664 nm, and pigment concentrations were calculated using Lichtenthaler and Buschmann (2001) equations.

For the determination of TPC, foliage samples were prepared in 96-well microplates, where 10 µL of the extract was mixed with 50 µL of Folin–Ciocalteu reagent (1:9 w/v in water) (VWR International GmbH, Vienna, Austria) (modified methodology by Beniušytė et al., 2023). The microplate was incubated for 5 min, and then 40 µl of 10% Na_2_CO_3_ was added. After one hour of incubation in the dark, the sample absorption at a wavelength of 725 nm was measured. Gallic acid (>98%, Carl Roth GmbH+Co. KG, Karlsruhe, Germany) was used for the calibration curve. TPC was calculated as gallic acid equivalent per gram of fresh weight (FW) foliage, as shown in Equation 1.


(1) TPC (mg/g) = (C × V)/m

where C is the concentration obtained from the calibration curve (mg/ml); V is the extract volume (ml); m is the weight of fresh biomass extracted (g).

The TFC was determined by forming a flavonoid-Al(III) complex (Chang et al., 2002). Sample absorption at a wavelength of 415 nm was measured. Quercetin (>98%, Cayman Chemical Company, Ann Arbor, USA) was used for the calibration curve. As in the Equation 1, TFC was calculated as quercetin equivalent per gram of FW foliage (mg/g).

For the TSS determination, the supernatant was mixed with 0.1% anthrone reagent (CarlRoth, Karlsruhe, Germany) and heated at 90°C for one hour (Agro-LAB termostating TFC 200, Venice, Italy). The absorption was measured at 620 nm. Glucose was used to create the calibration curve. TSS was calculated as quercetin equivalent per gram of FW foliage (mg/g).

For MDA determination, a modified methodology was used with 50 µL of the extract mixed with 130 μL of the reaction mixture, which was prepared by combining 20 % tri-chloroacetic acid (Molar chemicals Kft, Hungary) and 0.5 % (w/v) of thiobarbituric acid (Alfa Aesar, Germany) (Hodges et al., 1999). The obtained mixture was incubated at 95 °C for 30 min, and after incubation, it was cooled. The absorbance was measured at 440 nm, 532 nm, and 600 nm. The final quantity of MDA was calculated per gram of FW foliage using the Equation (2):


(2) MDA (nmol/g) = 6.45 × A532 − A600 − 0.56 × A440

where A_532_ – absorption value at 532 nm wavelength; A_600_ – absorption value at 600 nm wavelength; A_450_ – absorption value at 440 nm wavelength.

### Statistical analysis

Group means and standard errors (SE) were calculated using Microsoft Excel, while statistical analyses were performed with SPSS software (IBM, version 28.0.1.1). The Shapiro-Wilk test indicated that the data did not follow a normal distribution. Therefore, the Mann-Whitney U test, a non-parametric alternative suitable for independent group comparisons without normality assumptions, was used to assess differences between the control and PM treatment groups. Statistical significance was set at *p* ≤ 0.05.

## Results

### Response of stem growth and seedling biomass to simulated particulate matter

**Figure 1:**
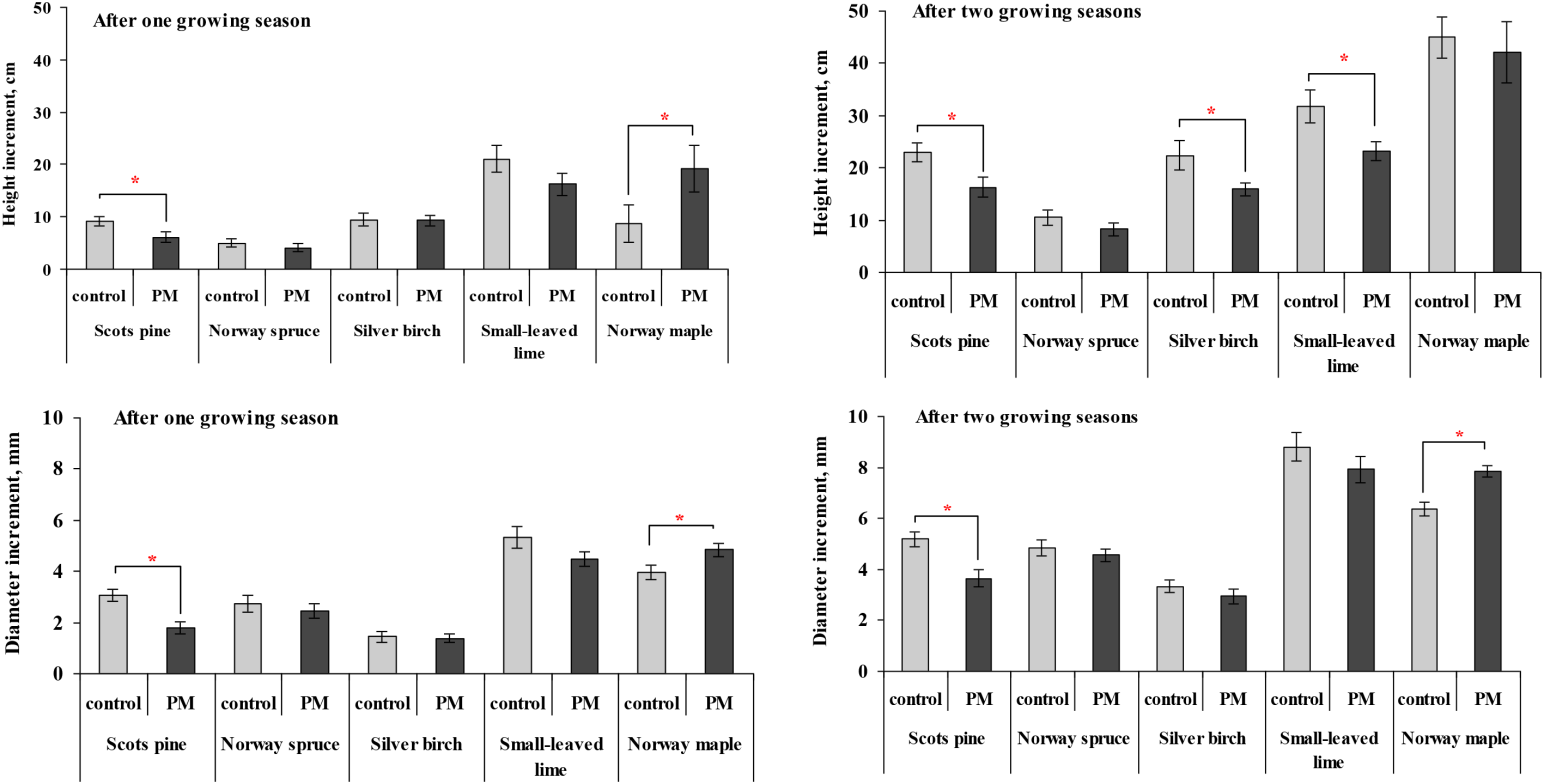
Comparison of the mean (± SE) increment in stem height (cm) and diameter (mm) of seedlings between the control and particulate matter (PM) treatment groups after one and two growing seasons. Statistical significance was determined using the Mann-Whitney U test (*p* ≤ 0.05). Asterisks (*) indicate significant differences from the control within the same species.

The PM significantly reduced Scots pine growth parameters (**Figure 1**). Compared to the control, Scots pine seedling height increment was 1.7–1.9 times lower, and diameter increment was 1.4–1.7 times lower after one and two growing seasons of exposure to PM pollution. Similarly, a significantly lower height increment was observed in silver birch and small-leaved lime seedlings after two growing seasons of exposure to PM pollution. Norway maple showed the opposite trend, with a greater height increment after one season of exposure to PM and a greater diameter increment in both seasons.

**Figure 2:**
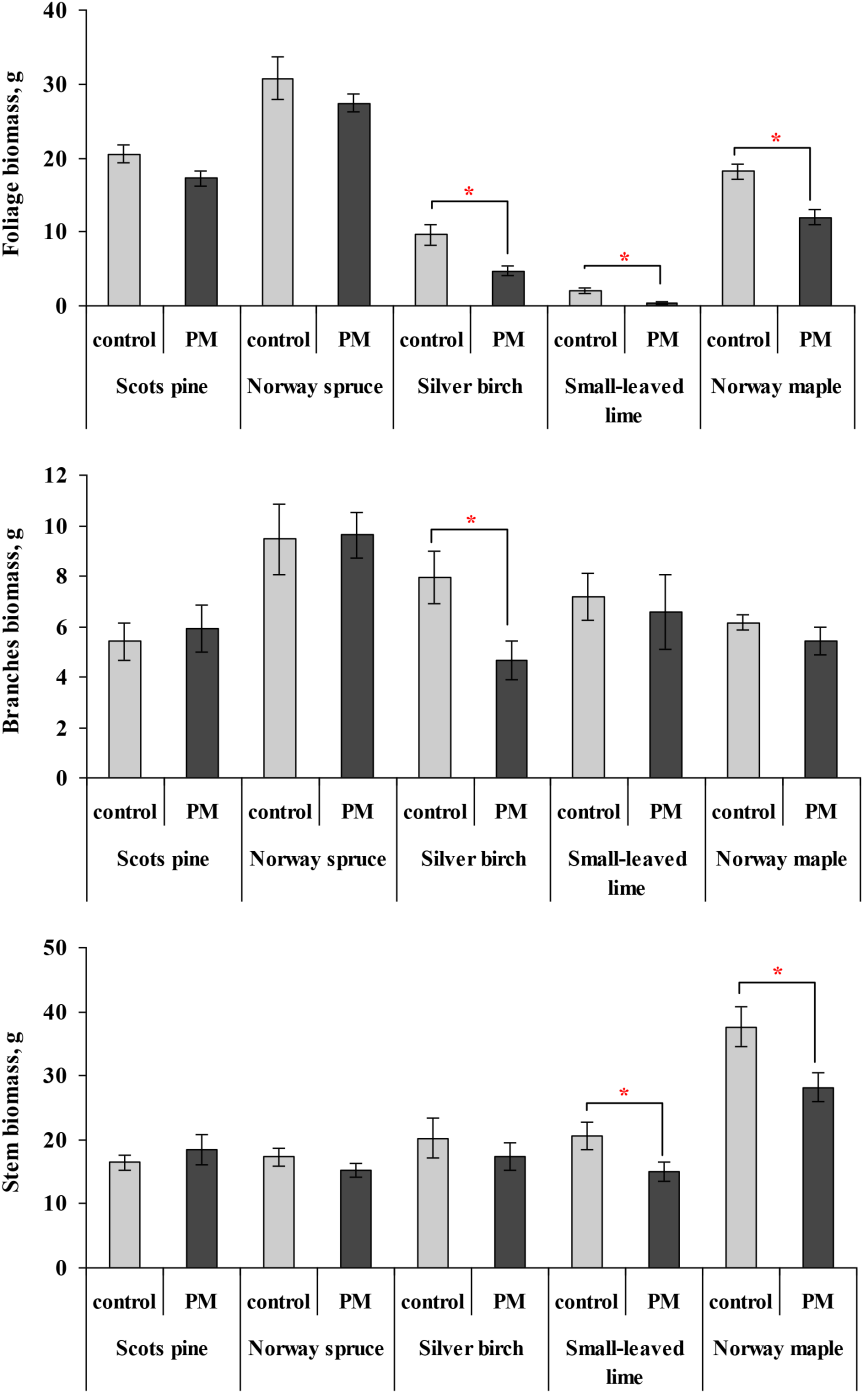
Comparison of the mean (± SE) biomass (grams of dry weight per seedling) of foliage, branches and stems in seedlings between the control and particulate matter (PM) treatment groups after two growing seasons. Statistical significance was determined using the Mann-Whitney U test (*p* ≤ 0.05). Asterisks (*) indicate significant differences from the control within the same species.

The PM treatment significantly reduced the foliage biomass of silver birch, Norway maple, and small-leaved lime seedlings after two growing seasons of PM exposure (**Figure 2**). Furthermore, silver birch had lower branch biomass, while small-leaved lime and Norway maple seedlings showed lower stem biomass.

### Response of biochemical parameters in seedling foliage to simulated particulate matter

**Figure 3:**
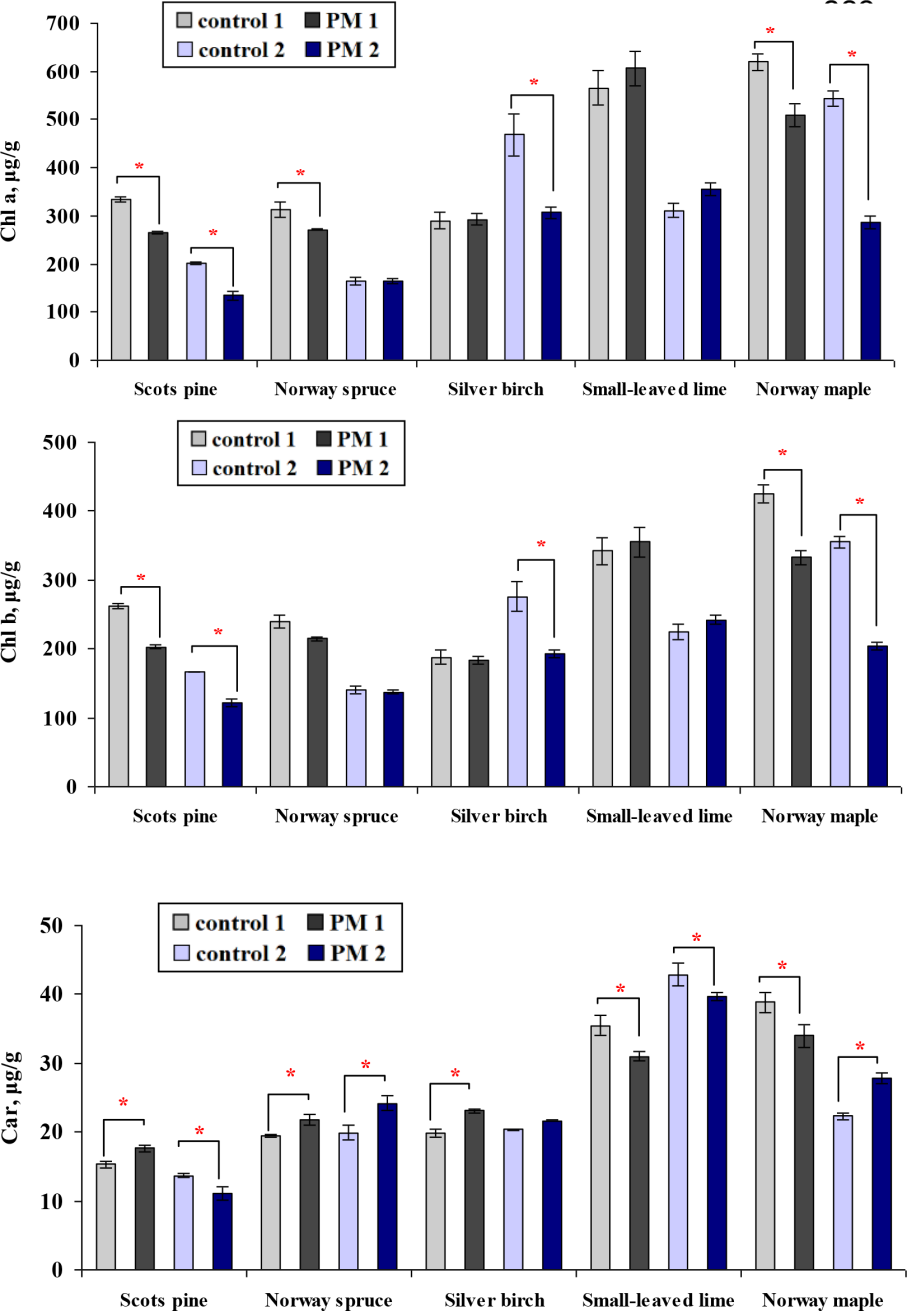
Comparison of the mean (± SE) concentrations of photosynthesis pigments (Chl a and b, µg/g) and carotenoids (Car, µg/g) in seedlings between the control and particulate matter (PM) treatment groups after one (control 1; PM 1) and two (control 2; PM 2) growing seasons. Statistical significance was determined using the Mann-Whitney U test (*p* ≤ 0.05). Asterisks (*) indicate significant differences from the control within the same species.

After one growing season, the PM treatment significantly reduced Chl a concentration for Scots pine, Norway spruce, and Norway maple and reduced Chl b concentrations for Scots pine and Norway maple (**Figure 3**). Two years after PM treatment, lower concentrations of pigments Chl a and Chl b remained for Scots pine and Norway maple and were also observed for silver birch. The response in the Car content was more variable for all species.

Because of species-specific growth and biochemical patterns and seasonal variability in meteorological conditions, comparisons across the two seasons could be made only by evaluating the differences between PM-treated and control seedlings. Silver birch seedlings responded to PM exposure with reductions in Chl a and Chl b only in the second season, compared to the first season (**Figure 3**). In contrast, Norway maple seedlings showed a decrease in Car content during the first season, followed by a significant increase in the second season.

**Figure 4:**
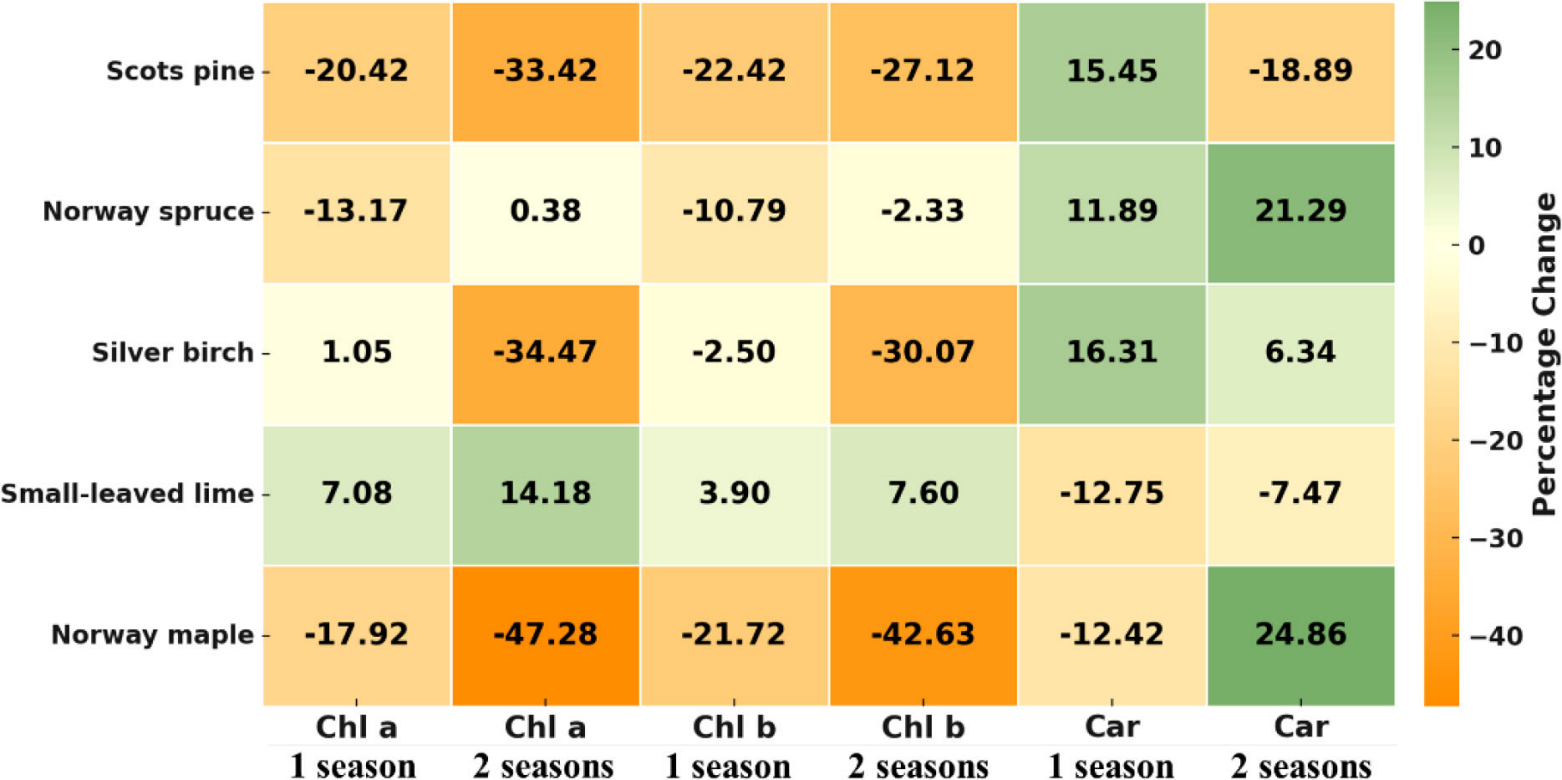
Heatmap of percentage changes in Chlorophyll a (Chl a), Chlorophyll b (Chl b) and Carotenoids (Car) due to PM exposure across different species and seasons. Significant decreases in orange, little to no change in white/yellow and slight increases in green.

Both pigments, Chl a and Chl b, showed negative percentage changes under PM exposure (**Figure 4**). For example, for Scots pine, Chl a decreased by about 20% after one season and by about 33% after two seasons. Chl b also showed declines in both seasons. Like Scots pine, Chl a and Chl b pigments responded similarly in silver birch and Norway maple. The opposite response was observed for small-leaved lime, with Chl a increasing by 7% and 14% in the first and second seasons, respectively, and Chl b increasing by 4% and 8% in the first and second seasons, respectively. Evaluating the pigment Chl a and Chl b change over two growing seasons, the species can be ranked from the largest decrease to the most significant increase: Norway maple, silver birch, Scots pine, Norway spruce and Small-leaved lime. However, the carotenoid response to PM differed, with species ranked as follows: Scots pine, small-leaved lime, silver birch, Norway spruce and Norway maple.

**Figure 5:**
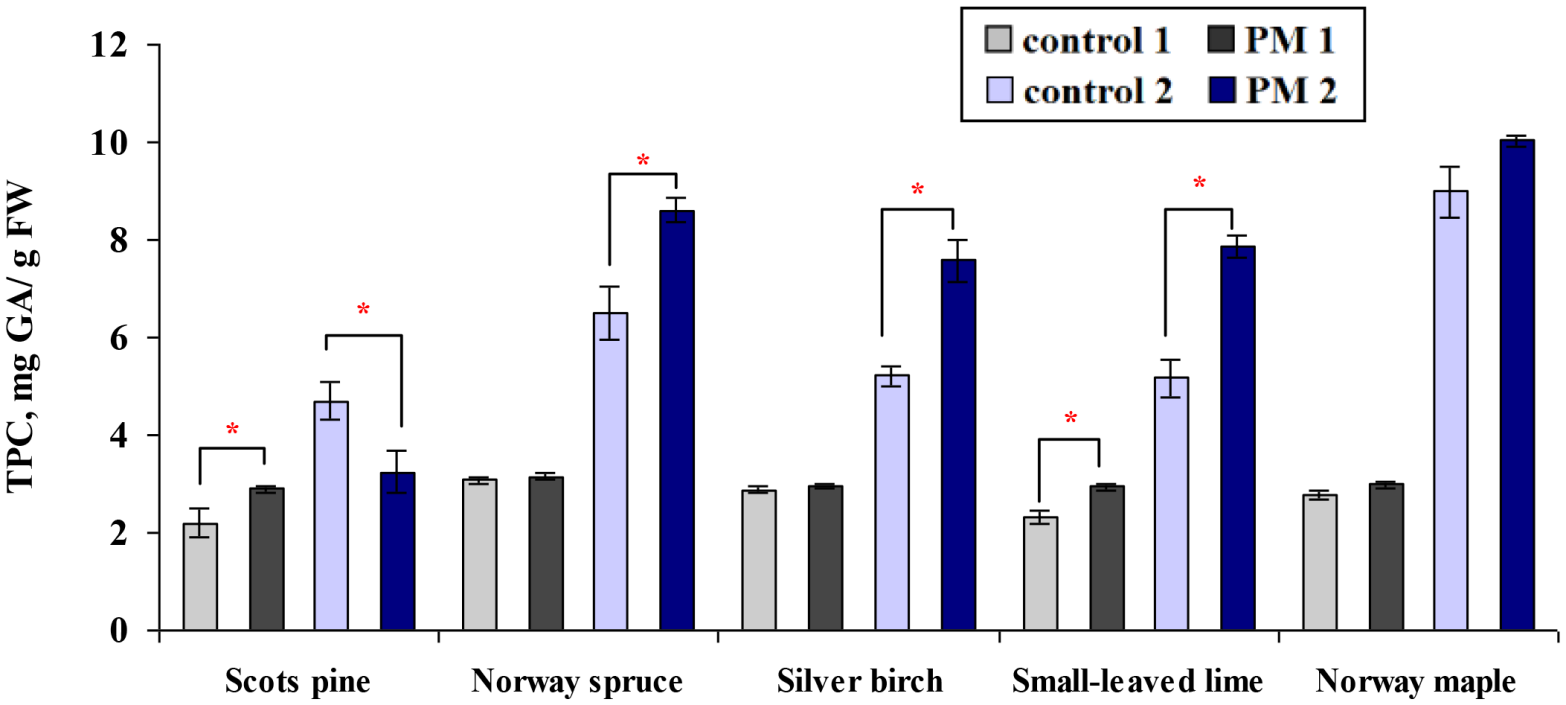
Comparison of the mean (± SE) content of total polyphenol (TPC, mg per gram of fresh weight (FW) foliage) in seedlings between the control and particulate matter (PM) treatment groups after one (control 1; PM 1) and two (control 2; PM 2) growing seasons. Statistical significance was determined using the Mann-Whitney U test (*p* ≤ 0.05). Asterisks (*) indicate significant differences from the control within the same species.

After one growing season, the TPC in the foliage of Scots pine and small-leaved lime seedlings exposed to PM increased significantly, by 1.3 times compared to the control (**Figure 5**). After two seasons, Norway spruce, silver birch, and Small-leaved lime seedlings showed TPC levels that were 1.3 to 1.5 times higher than the control. While Scots pine responded oppositely, TPC was reduced compared to the control after two seasons.

Across the two seasons, PM exposure significantly increased TPC content only in the second season for Norway spruce and silver birch seedlings (**Figure 5**). Scots pine seedlings showed a significant increase in TPC content during the first season, but it decreased in the second season.

**Figure 6:**
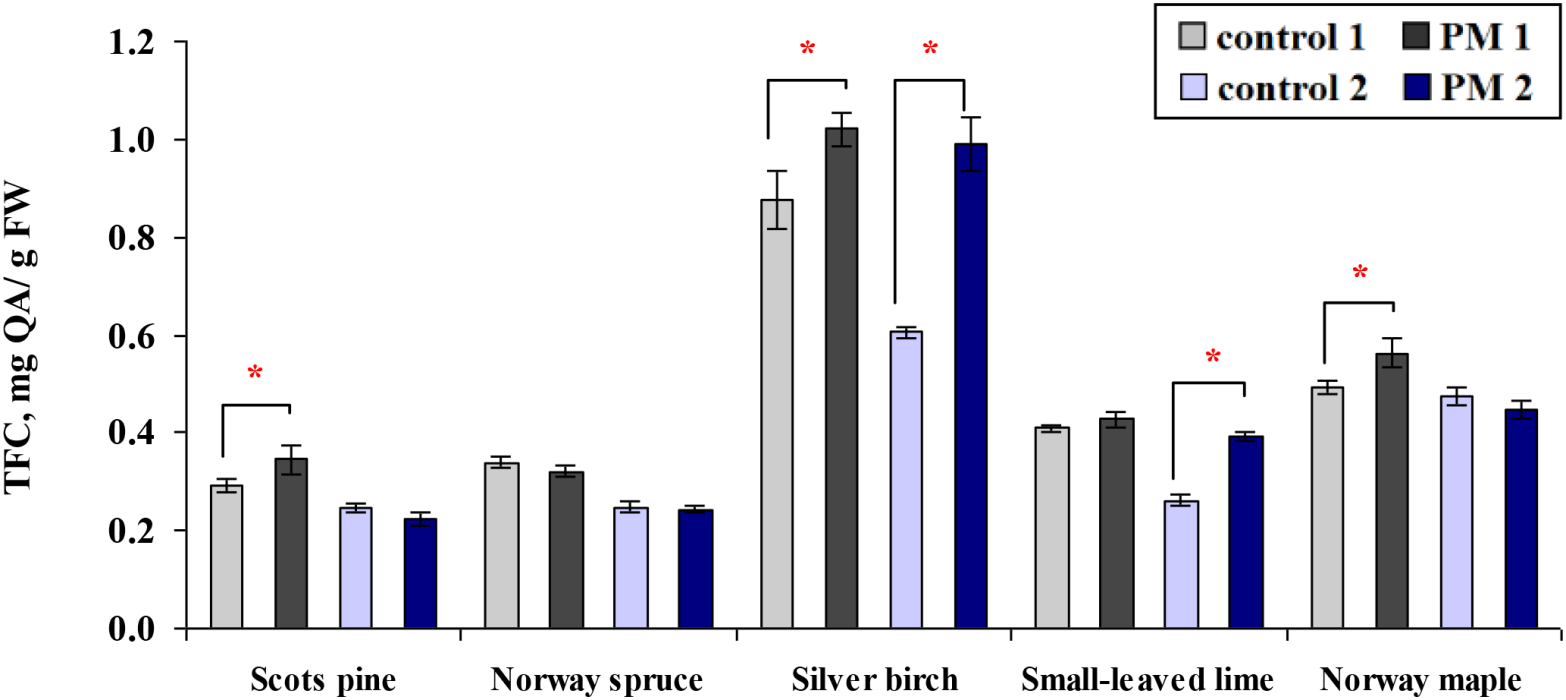
Comparison of the mean (± SE) content of total flavonoid (TFC, mg per gram of fresh weight (FW) foliage) in seedlings between the control and particulate matter (PM) treatment groups after one (control 1; PM 1) and two (control 2; PM 2) growing seasons. Statistical significance was determined using the Mann-Whitney U test (*p* ≤ 0.05). Asterisks (*) indicate significant differences from the control within the same species.

In the foliage of Scots pine, silver birch and Norway maple seedlings exposed to PM, TFC increased by 1.2 times after one season (**Figure 6**). After two seasons, TFC levels were 1.5–1.6 times higher in silver birch and small-leaved lime seedlings. Across the two seasons, PM exposure significantly increased TFC content in the first season for Scots pine and Norway maple seedlings, whereas small-leaved lime seedlings showed a significant increase only in the second season (**Figure 6**).

**Figure 7:**
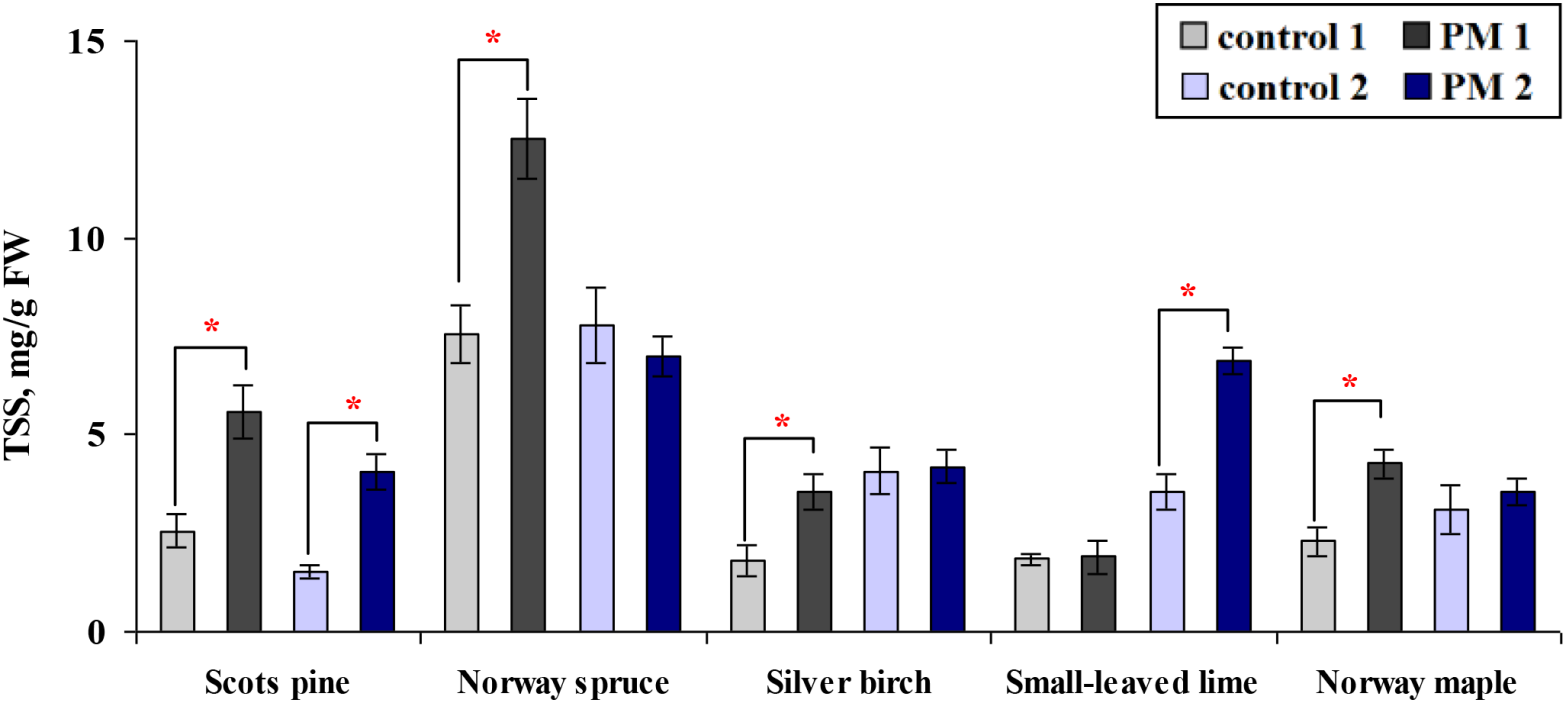
Comparison of the mean (± SE) content of total soluble sugar (TSS, mg per gram of fresh weight (FW) foliage) in seedlings between the control and particulate matter (PM) treatment groups after one (control 1; PM 1) and two (control 2; PM 2) growing seasons. Statistical significance was determined using the Mann-Whitney U test (*p* ≤ 0.05). Asterisks (*) indicate significant differences from the control within the same species.

The mean content of TSS increased by 1.7 to 2.2 times in the seedlings of all species, except for the small-leaved lime exposed to PM after the first growing season (**Figure 7**). After the second season, the TSS content was 2.7 times higher in Scots pine seedlings and 1.9 times higher in small-leaved lime compared to the control group. Across the two seasons, PM exposure increased TSS content in Norway spruce, silver birch, and Norway maple seedlings only in the first season, whereas small-leaved lime seedlings showed an increase only in the second season (**Figure 7**).

**Figure 8:**
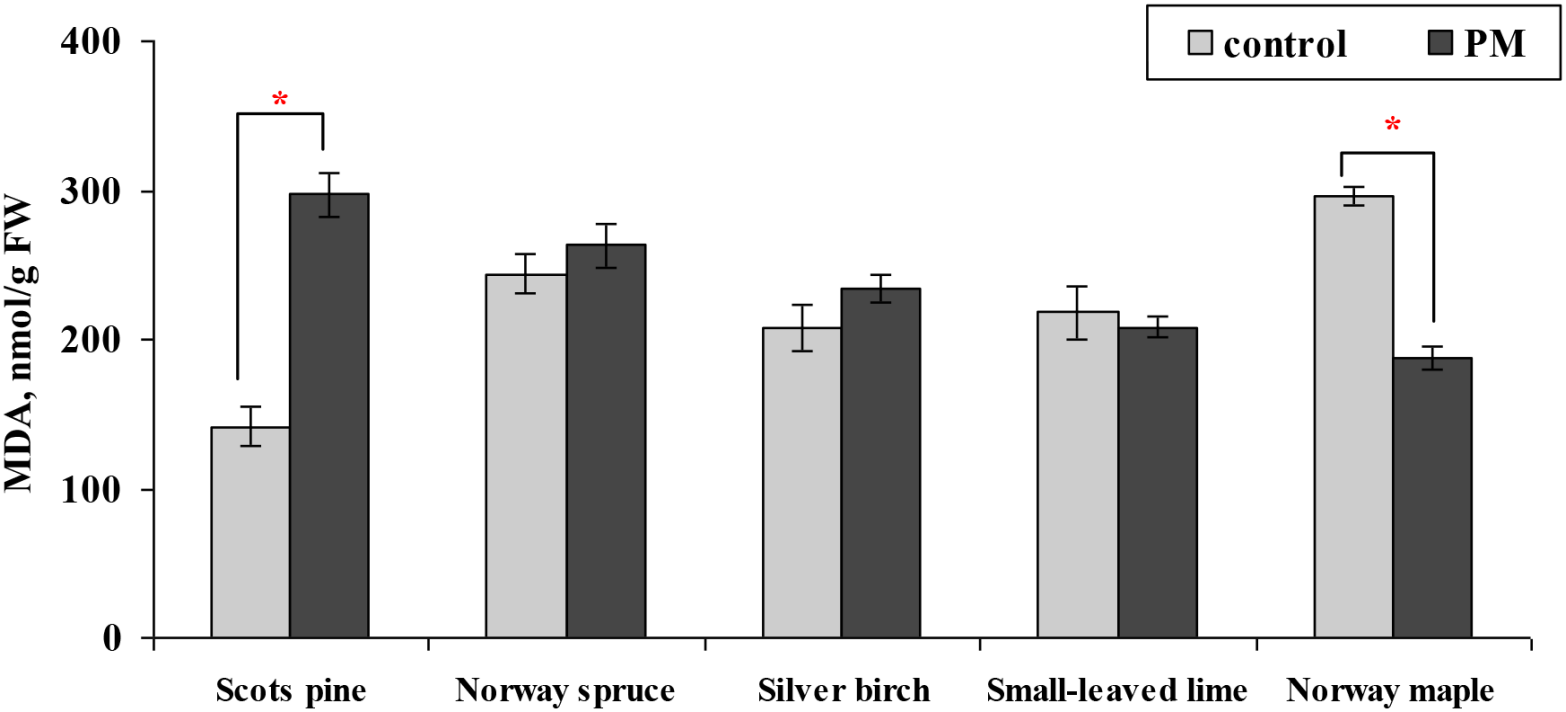
Comparison of the mean (± SE) concentration of malondialdehyde (MDA, nmol per gram of fresh weight (FW) foliage) in the foliage of seedlings between the control and particulate matter (PM) treatment groups after two growing seasons. Statistical significance was determined using the Mann-Whitney U test (*p* ≤ 0.05). Asterisks (*) indicate significant differences from the control within the same species.

The mean MDA levels significantly increased by 2 times for Scots pine needles, and in the opposite direction, the levels decreased 1.6 times for Norway maple leaves, and no changes were found for other species (**Figure 8**).

### Integrated analysis of growth and biochemical responses in tree seedlings following PM exposure

**Figure 9:**
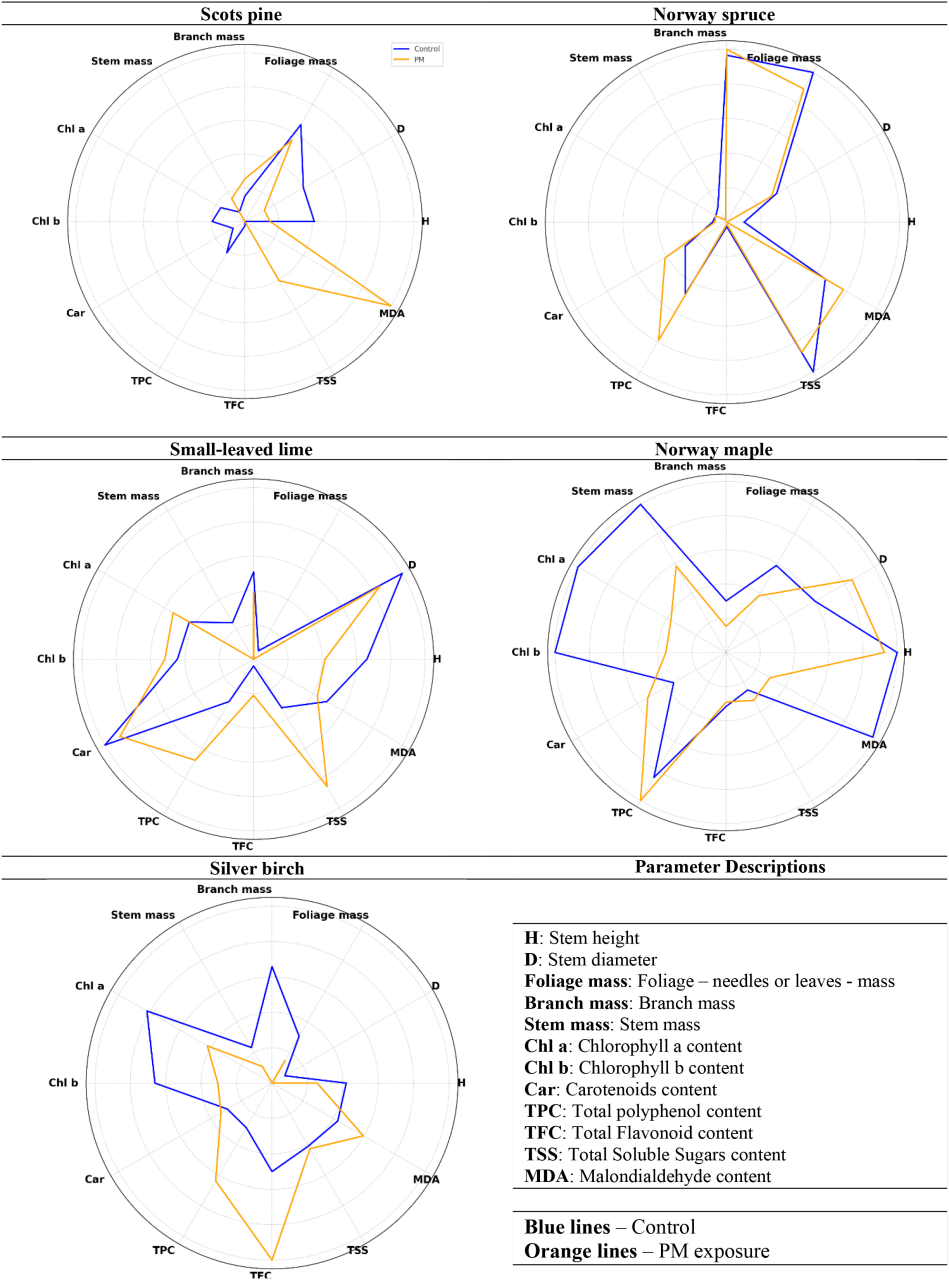
Radar analysis of growth and biochemical responses of tree species: control vs particulate matter (PM) effect, two years after PM treatment.

Two seasons after PM exposure, Scots pine, Norway spruce, silver birch, small-leaved lime, and Norway maple seedlings showed varying changes in growth and biochemical parameters compared to the control group (**Figure 9**). For Scots pine seedlings, decreases of 30–46% were observed in H, D, Chl a, Chl b, and TPC, while TSS and MDA increased 2.7 times and 2.1 times, respectively. Norway spruce seedlings showed a 39% decrease in H, 11–12% decreases in foliage mass, stem mass, and TSS, and 20–30% increases in Car and TPC. Silver birch seedlings responded with a 50% decrease in foliage mass, 29–41% decreases in H, branch mass, Chl a, and Chl b, and 45–62% increases in TPC and TFC. Small-leaved lime seedlings had 85% decreases in foliage mass and 27% in stem mass, with 94% increases in TSS and 50–52% increases in TPC and TFC. Norway maple seedlings showed 47–50% decreases in Chl a, 35–43% decreases in Chl b, MDA, and foliage mass, a 25% decrease in stem mass, and approximately 23–25% increases in Car and D.

Correlation analysis of PM-treated trees over two seasons showed species-specific patterns in biochemical and growth responses (**Supplementary Table 1**). All species showed a very strong positive correlation between Chl a and Chl b (r = 0.96–1.00). The Chl a and Car showed a positive correlation in Scots pine and small-leaved lime; however, a negative correlation was observed in Norway spruce and Norway maple. There were positive correlations between TPC and chlorophylls, and between TPC and TFC (r = 0.85–0.88) for silver birch, and between TPC and TSS (r = 0.71) in Norway spruce. The correlations between TFC and chlorophylls were positive in both coniferous species and negative in Norway maple. The TFC and Car positively correlated in Scots pine and Norway maple, but negatively in Norway spruce. Specifically for Norway spruce, TSS content was negatively related to chlorophylls and positively to Car and TPC. Negative correlations between TSS and Car were also found in silver birch and small-leaved lime. The correlation analyses of growth parameters, such as H and D increments, showed no relations with other parameters, except for an interrelation in small-leaved lime. Seedling biomass elements showed species-specific relations with biochemical parameters. For Scots pine seedlings, positive correlations were found between leaf and stem masses with Chl a, Chl b, Car, TFC, and branch mass; also, between branch mass and Car. A negative correlation was found between branch mass and MDA level. In Norway spruce, there were positive correlations between stem mass with chlorophylls and leaf mass, while negative correlations were found with Car and TSS.

In deciduous tree species, silver birch seedlings showed positive correlations among leaf mass, branch mass, and stem mass. In small-leaved lime, stem mass was positively correlated with branch mass, while in Norway maple seedlings, a positive correlation was observed between stem mass and leaf mass.

## Discussion

The initial aim of this study was to assess the effects of simulated PM pollution on the growth, biomass allocation, and biochemical responses of seedlings from five tree species: Scots pine, Norway spruce, silver birch, small-leaved lime, and Norway maple. These species are ecologically significant in the region and are frequently planted in urban areas where they are consistently exposed to high levels of air pollution. By examining the complex responses to controlled PM exposure, we aimed to better understand species-specific strategies and their potential applicability for planting in polluted urban areas. PM-tolerant plants should be a priority when growing in urban areas (Rai, 2016). Plant adaptation to environmental stress involves short-term responses that can cause long-term changes (Dineva, 2004).

The first question in this study sought to determine the species-specific changes in coniferous and deciduous tree seedling growth and biomass one to two years after PM exposure. The study showed that PM treatment significantly reduced seedling height increment in silver birch, small-leaved lime, and height and diameter increment of Scots pine seedlings following two years of PM exposure. Meanwhile, Norway maple showed increased diameter growth. Another important finding was that the PM treatment significantly reduced foliage biomass in silver birch, Norway maple, and small-leaved lime after two growing seasons. Additionally, species-specific reductions were observed in other biomass components: silver birch responded in lower branch biomass, while stem biomass declined in small-leaved lime and Norway maple. Changes in tree growth may not be immediately apparent in the short term, while high levels of PM over extended periods can cause morphological and physiological damage to plants, primarily because PM often clogs the stomatal pores, reducing carbon dioxide (CO_2_) flux and thus diminishing photosynthetic activity (Rai, 2016; de Barros Ruas et al., 2022). Changes in the photosynthetic pigment chlorophyll, which is an essential component of plant metabolism, are commonly used to assess the effects of air pollutants on plants (Prusty et al., 2005; Rai, 2016). Variations in chlorophyll levels can influence a plant’s physiological, biochemical, and morphological characteristics (Verma and Singh, 2006). Initial PM effects on chlorophyll are related to the negative impact of photosynthesis, which primarily acts through a shading effect. Accumulated particles absorb and scatter sunlight, reducing light availability to chloroplasts and thus lowering photosynthetic efficiency (Przybysz et al., 2014; Leghari et al., 2014; Popek et al., 2017).

Łukowski et al. (2020) conducted an experimental study on how PM pollution from various sources - cement, construction, and roadside - affects the growth and ecophysiology of saplings of *Betula pendula*, *Tilia cordata* and *Quercus robur*. They found that PM accumulation on leaves reduced the efficiency of the photosynthetic apparatus and disrupted normal plant functioning. Among these species, the photosynthetic rate in *Betula pendula* was negatively affected, while in *Tilia cordata*, the length of the leader shoot increased. Previous studies have also demonstrated that PM adversely affected the photosynthetic apparatus and negatively influenced plant growth (Leghari et al., 2014). This negative effect may be attributable to a correlation between biochemical parameters and the growth and development of plants (Leghari et al., 2014; Rai, 2016). Prasad and Inamdar (1990) observed that cement-derived PM reduced the height of the non-woody plant black gram (*Vigna mungo* (L.) Hepper), likely due to decreased photosynthesis and elevated respiration rates in response to environmental stress. In our study, we found that pigment and antioxidant responses to PM exposure were species-specific, addressing the second question of this study. Simulated PM pollution significantly reduced Chl a and b concentrations in most tree species, particularly Scots pine, Norway maple, and silver birch, with declines persisting over two growing seasons. Scots pine showed the most substantial decline, with Chl a decreasing by up to 33%. Most often, chlorophyll concentrations are lower under PM stress than in the control (Rai and Panda, 2014; Gupta et al., 2016; Rai, 2016; Hariram et al., 2018). As demonstrated by Prusty et al. (2005), high PM levels reduce chlorophyll content in plant cells, and Popek et al. (2017) showed that Chl a level can also be changed. Consequently, the reductions in chlorophyll level directly reduce plant growth.

While many species showed reduced chlorophyll content under pollution stress, our study found that small-leaved lime showed the opposite trend, with chlorophyll levels increasing under PM exposure. This suggests a species-specific response to air pollutants. Consistent with our results, Abriha-Molnár et al. (2024) and Tuzhilkina (2009) found elevated chlorophyll levels in deciduous species – common hackberry (*Celtis occidentalis* L.) and conifers – Scots pine and Siberian spruce (*Picea obovata* Ledeb.), respectively, when exposed to moderate PM pollution. The compensatory mechanisms explained higher chlorophyll levels in silver birch growing in areas with higher pollution (Petrova, 2011).

The Car content responses to PM were found to be more variable: Scots pine showed the most pronounced changes in Car content, while Norway maple was least affected. This indicates that Car dynamics under PM stress are not necessarily aligned with chlorophyll responses and may reflect different protective or stress-related physiological processes.

The third question in this study was to analyze the potential activation of antioxidant defenses in response to PM stress. This study revealed that exposure to PM generally increased antioxidant compounds (TPC and TFC) in seedling foliage, but species responses varied over time. Small-leaved lime showed consistent TPC increases across both seasons, while Scots pine initially showed elevated TPC but declined after two seasons. TFC levels increased in several species, with silver birch and small-leaved lime showing the most apparent increase after two seasons, indicating a potential activation of antioxidant defenses in response to PM stress. Loponen et al. (2001) found no change in total phenolics in *Betula* leaves, though gallic acid declined, likely due to disrupted biosynthetic pathways. Kovalikova et al. (2021) reported increased total phenols in birch, oak, and maple buds under medium to high pollution, but phenolic acids were highest in low-pollution areas. Lahiri and Krishna (2024) found that foliar phenols in *Moringa oleifera* Lam. decreased under moderate and severe air pollution. Individual phenolics responded differently to pollution, which may explain the species-specific reduction in foliar phenols.

Exposure to PM and other air pollutants did not affect the flavonoid content in saplings and indicated a stable flavonoid content under varying environmental and pollution conditions. It was also noted that the flavonoid content decreased with increasing pollution levels in *Psidium guajava* L.*, Murraya konegii* L.*, and Moringa oleifera* (Lahiri and Krishna, 2024).

Our experiment did not reveal substantial differences in TSS content responses to PM among species. Most notably, following two growing seasons, the most significant increase in TSS content, 2.7 times, was observed in Scots pine, while a 1.9 times increase was observed in small-leaved lime. In the study by Tian et al. (2024), which investigated the effects of varying dust exposure durations, leaf TSS content initially increased and declined over time. Previous studies have demonstrated that urban environments can have an adverse impact on tree carbohydrate reserves. Banerjee et al. (2021), in their assessment of *Ficus religiosa* L., *Anthocephalus cadamba* (Roxb.) Bosser, *Lagerstroemia* sp*eciosa* (L.) Pers., and *Cassia siamea* (Lam.) Irwin et Barneby in West Bengal, India, reported that sensitive species resulted in significantly lower TSS levels under air pollution due to the deformation of chlorophyll molecules. Similarly, Rai (2016) showed the decline in TSS levels in pollution-sensitive plants due to the blockage of stomatal pores by PM, impairing light penetration and reducing photosynthetic efficiency. While our data were obtained from very young tree seedlings after intense PM exposure, the short-term response may have been more specific than that observed in mature trees.

Furthermore, lipid peroxidation, estimated by the amount of MDA, increased in Scots pine (2.1 times) but decreased in Norway maple (37%). Exposure to PM has been linked to increased oxidative damage in plants. Due to MDA accumulation under stress conditions, MDA levels indicate cellular damage in plants, with elevated levels typically reflecting more significant physiological injury (Morales and Munné-Bosch, 2019; Alché, 2019). Elevated MDA levels were obtained in tree species capable of accumulating large amounts of PM, such as *Sophora japonica* L., *Euonymus japonicus* Thunb., and *Ulmus pumila* L. (Zhang et al., 2020a). The increase in MDA levels in urban trees indicates that leaf-surface PM deposition contributes to oxidative stress in plants (Chaudhary and Rathore, 2018). These authors also indicated that dust deposition negatively influenced the foliage dry weight and photosynthetic pigments. The changes in MDA under PM exposure were also supported by Lhotská et al. (2022), who observed an approximately 24% increase in MDA content in lettuce after applying soil dust PM.

In the final step of this study, integrated physiological and growth responses were revealed. Clear links were found between Chl a and Chl b for all species. The correlations between chlorophylls and Car were positive for Scots pine and small-leaved lime, but negative for Norway spruce and Norway maple. The interrelationships among chlorophylls, carotenoids, and total sugars have been demonstrated in prior research (Prasad and Inamdar, 1990). Positive correlations were found between TPC and TFC only in silver birch. Importantly, MDA showed a negative correlation only with Scots pine branch mass, suggesting that oxidative stress limited the growth of Scots pine seedlings under PM exposure.

The seedling responses may be attributed to the varying activation of defense mechanisms, including stress hormones, proteins, and antioxidants, which were not evaluated in our study. Previous studies suggest that antioxidants protect the photosynthetic apparatus against oxidative stress, enhancing photosynthetic pigment content (Agathokleous et al., 2020). These processes support the idea of environmental hormesis, in which low-dose stressors in urban areas can induce beneficial effects (Erofeeva, 2020; Jalal et al., 2021). Norway maple, known for its tolerance to various environmental stressors, showed increased carotenoid levels, reduced MDA content - indicating decreased lipid peroxidation - and enhanced growth two years after PM treatment. These responses are consistent with hormetic effects. Alternatively, PM pollution, which was artificially simulated approximately once per week during the vegetation period, could have a specific response compared to persistent, low-level pollution throughout the year.

It is important to note that the applicability of our findings is constrained by certain limitations. Because the morphological and physiological characteristics of young seedlings differ from those of adult trees (e.g., Thomas and Winner, 2002; Mediavilla and Escudero, 2003; Zhang et al., 2020b), the responses observed in this study cannot be directly extrapolated to adult trees. Our study artificially simulated the elevated PM exposure that trees may experience in heavily polluted urban environments, but caution is needed when projecting these results to real-world PM depositions.

## Conclusion

This study evaluated growth, biomass, and biochemical responses of silver birch (*Betula pendula*), small-leaved lime (*Tilia cordata*), Norway maple (*Acer platanoides*), Scots pine (*Pinus sylvestris*) and Norway spruce (*Picea abies*) seedlings under simulated particulate matter (PM) exposure. Results demonstrated species-specific responses. Norway maple and small-leaved lime showed the highest resilience, maintaining growth and activating defense mechanisms under PM pollution stress. Silver birch demonstrated moderate tolerance, with biochemical compensation, despite growth suppression. Norway spruce showed physiological imbalance and reduced growth. Scots pine was the most sensitive, with significant growth reduction, pigment loss, and oxidative stress.

Based on the findings, Norway maple and small-leaved lime are recommended for urban planting in areas with high PM levels, while silver birch and Norway spruce may be suitable in areas with moderate PM pollution. Meanwhile, Scots pine was sensitive to PM exposure at the seedling stage and may be less suitable for urban environments with high PM levels. Limitations include the use of seedlings and the short-term effects, which may underestimate the resilience of adult trees.

## Data Availability

The original contributions presented in the study are included in the article/**Supplementary Material**. Further inquiries can be directed to the corresponding author.

## Supplementary material

Supplementary Table 1 Species-specific correlation of biochemical and growth parameters under PM exposure. The asterisk (*) next to a correlation value indicates statistical significance at *p* ≤ 0.05.
