# Supplementary material for: Species-specific responses of young deciduous and coniferous trees to simulated particulate matter
Source: Front Plant Sci. 2025 Oct 8;16:1622995. doi: 10.3389/fpls.2025.1622995 (PMC12540493; doi:10.3389/fpls.2025.1622995)
Supplement: Supplementary Table 1 — Species-specific correlation of biochemical and growth parameters under PM exposure. The asterisk (*) next to a correlation value indicates statistical significance at p ≤ 0.05. [file Table1.docx]

**Supplementary material**

SUPPLEMENTARY TABLE 1. Species-specific correlation of biochemical and growth parameters under PM exposure. Note: the asterisk (*) next to a correlation value indicates statistical significance at *p* ≤ 0.05.

|  | **Chl a** | **Chl b** | **Car** | **TPC** | **TFC** | **TSS** | **MDA** | **Leaf**  **mass** | **Branch**  **mass** | **Stem**  **mass** | **H** | **D** |
| --- | --- | --- | --- | --- | --- | --- | --- | --- | --- | --- | --- | --- |
|  | **Scots pine** | | |  |  |  |  |  |  |  |  |  |
| **Chl a** | 1.00 | 1.00* | 0.91* | 0.30 | 0.93* | 0.59 | -0.17 | 0.71* | 0.60 | 0.74* | -0.12 | 0.35 |
| **Chl b** | 1.00* | 1.00 | 0.89* | 0.33 | 0.92* | 0.64 | -0.12 | 0.67* | 0.56 | 0.71* | -0.12 | 0.34 |
| **Car** | 0.91* | 0.89* | 1.00 | 0.42 | 0.95* | 0.46 | -0.54 | 0.74* | 0.80* | 0.84* | -0.23 | 0.34 |
| **TPC** | 0.30 | 0.33 | 0.42 | 1.00 | 0.49 | 0.58 | -0.16 | 0.16 | 0.02 | 0.01 | -0.48 | 0.09 |
| **TFC** | 0.93* | 0.92* | 0.95* | 0.49 | 1.00 | 0.51 | -0.38 | 0.67* | 0.58 | 0.72* | -0.40 | 0.33 |
| **TSS** | 0.59 | 0.64 | 0.46 | 0.58 | 0.51 | 1.00 | 0.24 | 0.17 | 0.07 | 0.16 | -0.01 | -0.06 |
| **MDA** | -0.17 | -0.12 | -0.54 | -0.16 | -0.38 | 0.24 | 1.00 | -0.36 | -0.68* | -0.51 | 0.36 | -0.21 |
| **Leaf mass** | 0.71* | 0.67* | 0.74* | 0.16 | 0.67* | 0.17 | -0.36 | 1.00 | 0.75* | 0.57 | 0.10 | 0.46 |
| **Branch mass** | 0.60 | 0.56 | 0.80* | 0.02 | 0.58 | 0.07 | -0.68* | 0.75* | 1.00 | 0.87* | 0.13 | 0.27 |
| **Stem mass** | 0.74* | 0.71* | 0.84* | 0.01 | 0.72* | 0.16 | -0.51 | 0.57 | 0.87* | 1.00 | 0.03 | 0.12 |
| **H** | -0.12 | -0.12 | -0.23 | -0.48 | -0.40 | -0.01 | 0.36 | 0.10 | 0.13 | 0.03 | 1.00 | -0.40 |
| **D** | 0.35 | 0.34 | 0.34 | 0.09 | 0.33 | -0.06 | -0.21 | 0.46 | 0.27 | 0.12 | -0.40 | 1.00 |
|  | **Norway spruce** | |  |  |  |  |  |  |  |  |  |  |
| **Chl a** | 1.00 | 0.96* | -0.93* | -0.41 | 0.85* | -0.67* | 0.07 | 0.41 | 0.39 | 0.71* | -0.44 | -0.18 |
| **Chl b** | 0.96* | 1.00 | -0.98* | -0.63 | 0.75* | -0.78* | -0.11 | 0.44 | 0.51 | 0.74* | -0.51 | -0.20 |
| **Car** | -0.93* | -0.98* | 1.00 | 0.62 | -0.79* | 0.82* | 0.13 | -0.49 | -0.59 | -0.75* | 0.40 | 0.03 |
| **TPC** | -0.41 | -0.63 | 0.62 | 1.00 | -0.11 | 0.71* | 0.35 | -0.24 | -0.48 | -0.37 | 0.50 | 0.23 |
| **TFC** | 0.85* | 0.75* | -0.79* | -0.11 | 1.00 | -0.54 | 0.07 | 0.31 | 0.28 | 0.55 | 0.02 | 0.19 |
| **TSS** | -0.67* | -0.78* | 0.82* | 0.71* | -0.54 | 1.00 | 0.15 | -0.75* | -0.70* | -0.76* | 0.13 | -0.08 |
| **MDA** | 0.07 | -0.11 | 0.13 | 0.35 | 0.07 | 0.15 | 1.00 | 0.23 | -0.45 | 0.14 | -0.10 | -0.13 |
| **Leaf mass** | 0.41 | 0.44 | -0.49 | -0.24 | 0.31 | -0.75* | 0.23 | 1.00 | 0.57 | 0.83* | 0.02 | 0.15 |
| **Branch mass** | 0.39 | 0.51 | -0.59 | -0.48 | 0.28 | -0.70* | -0.45 | 0.57 | 1.00 | 0.54 | 0.05 | 0.27 |
| **Stem mass** | 0.71* | 0.74* | -0.75* | -0.37 | 0.55 | -0.76* | 0.14 | 0.83* | 0.54 | 1.00 | -0.24 | -0.17 |
| **H** | -0.44 | -0.51 | 0.40 | 0.50 | 0.02 | 0.13 | -0.10 | 0.02 | 0.05 | -0.24 | 1.00 | 0.62 |
| **D** | -0.18 | -0.20 | 0.03 | 0.23 | 0.19 | -0.08 | -0.13 | 0.15 | 0.27 | -0.17 | 0.62 | 1.00 |
|  | **Silver birch** | |  |  |  |  |  |  |  |  |  |  |
| **Chl a** | 1.00 | 0.99* | 0.55 | 0.85* | 0.51 | -0.27 | 0.66 | 0.27 | 0.48 | 0.42 | -0.34 | 0.09 |
| **Chl b** | 0.99* | 1.00 | 0.47 | 0.88* | 0.56 | -0.22 | 0.66 | 0.25 | 0.49 | 0.43 | -0.41 | 0.11 |
| **Car** | 0.55 | 0.47 | 1.00 | 0.46 | 0.49 | -0.69* | 0.37 | 0.21 | 0.25 | 0.26 | -0.02 | 0.16 |
| **TPC** | 0.85* | 0.88* | 0.46 | 1.00 | 0.85* | -0.12 | 0.66 | 0.10 | 0.36 | 0.39 | -0.54 | 0.17 |
| **TFC** | 0.51 | 0.56 | 0.49 | 0.85* | 1.00 | -0.28 | 0.52 | 0.06 | 0.26 | 0.29 | -0.51 | 0.34 |
| **TSS** | -0.27 | -0.22 | -0.69* | -0.12 | -0.28 | 1.00 | -0.13 | -0.42 | -0.50 | -0.31 | 0.14 | -0.16 |
| **MDA** | 0.66 | 0.66 | 0.37 | 0.66 | 0.52 | -0.13 | 1.00 | -0.18 | -0.12 | -0.21 | -0.11 | 0.07 |
| **Leaf mass** | 0.27 | 0.25 | 0.21 | 0.10 | 0.06 | -0.42 | -0.18 | 1.00 | 0.87* | 0.85* | 0.15 | 0.42 |
| **Branch mass** | 0.48 | 0.49 | 0.25 | 0.36 | 0.26 | -0.50 | -0.12 | 0.87* | 1.00 | 0.90* | -0.33 | 0.32 |
| **Stem mass** | 0.42 | 0.43 | 0.26 | 0.39 | 0.29 | -0.31 | -0.21 | 0.85* | 0.90* | 1.00 | -0.15 | 0.17 |
| **H** | -0.34 | -0.41 | -0.02 | -0.54 | -0.51 | 0.14 | -0.11 | 0.15 | -0.33 | -0.15 | 1.00 | -0.09 |
| **D** | 0.09 | 0.11 | 0.16 | 0.17 | 0.34 | -0.16 | 0.07 | 0.42 | 0.32 | 0.17 | -0.09 | 1.00 |
|  | **Small-leaved lime** | | | |  |  |  |  |  |  |  |  |
| **Chl a** | 1.00 | 0.97* | 0.96* | 0.38 | 0.44 | -0.63 | 0.48 | 0.44 | -0.13 | -0.48 | -0.25 | -0.01 |
| **Chl b** | 0.97* | 1.00 | 0.89* | 0.44 | 0.45 | -0.50 | 0.40 | 0.40 | -0.26 | -0.61 | -0.21 | 0.00 |
| **Car** | 0.96* | 0.89* | 1.00 | 0.27 | 0.42 | -0.68* | 0.45 | 0.42 | -0.00 | -0.37 | -0.17 | 0.08 |
| **TPC** | 0.38 | 0.44 | 0.27 | 1.00 | -0.39 | 0.13 | 0.50 | 0.28 | -0.32 | -0.52 | -0.13 | 0.06 |
| **TFC** | 0.44 | 0.45 | 0.42 | -0.39 | 1.00 | -0.56 | -0.37 | 0.09 | -0.45 | -0.31 | -0.20 | -0.09 |
| **TSS** | -0.63 | -0.50 | -0.68* | 0.13 | -0.56 | 1.00 | -0.35 | -0.33 | -0.08 | -0.09 | 0.48 | 0.51 |
| **MDA** | 0.48 | 0.40 | 0.45 | 0.50 | -0.37 | -0.35 | 1.00 | 0.47 | 0.39 | 0.19 | -0.55 | -0.35 |
| **Leaf mass** | 0.44 | 0.40 | 0.42 | 0.28 | 0.09 | -0.33 | 0.47 | 1.00 | -0.17 | -0.29 | -0.18 | -0.01 |
| **Branch mass** | -0.13 | -0.26 | -0.00 | -0.32 | -0.45 | -0.08 | 0.39 | -0.17 | 1.00 | 0.81* | 0.08 | 0.01 |
| **Stem mass** | -0.48 | -0.61 | -0.37 | -0.52 | -0.31 | -0.09 | 0.19 | -0.29 | 0.81* | 1.00 | -0.21 | -0.30 |
| **H** | -0.25 | -0.21 | -0.17 | -0.13 | -0.20 | 0.48 | -0.55 | -0.18 | 0.08 | -0.21 | 1.00 | 0.75* |
| **D** | -0.01 | 0.00 | 0.08 | 0.06 | -0.09 | 0.51 | -0.35 | -0.01 | 0.01 | -0.30 | 0.75* | 1.00 |
|  | **Norway maple** | | | | | | | | | | | |
| **Chl a** | 1.00 | 0.96* | -0.79* | -0.26 | -0.88* | -0.56 | -0.40 | -0.22 | -0.04 | -0.30 | 0.16 | 0.07 |
| **Chl b** | 0.96* | 1.00 | -0.61 | -0.04 | -0.72* | -0.42 | -0.36 | -0.17 | 0.16 | -0.17 | 0.31 | 0.18 |
| **Car** | -0.79* | -0.61 | 1.00 | 0.62 | 0.94* | 0.58 | 0.12 | 0.19 | 0.27 | 0.51 | 0.24 | 0.31 |
| **TPC** | -0.26 | -0.04 | 0.62 | 1.00 | 0.57 | 0.31 | 0.29 | 0.25 | 0.49 | 0.65 | 0.30 | 0.21 |
| **TFC** | -0.88* | -0.72* | 0.94* | 0.57 | 1.00 | 0.52 | 0.23 | 0.10 | 0.29 | 0.39 | 0.15 | 0.17 |
| **TSS** | -0.56 | -0.42 | 0.58 | 0.31 | 0.52 | 1.00 | 0.66 | 0.68* | 0.35 | 0.52 | 0.09 | 0.14 |
| **MDA** | -0.40 | -0.36 | 0.12 | 0.29 | 0.23 | 0.66 | 1.00 | 0.71* | 0.22 | 0.46 | -0.27 | -0.44 |
| **Leaf mass** | -0.22 | -0.17 | 0.19 | 0.25 | 0.10 | 0.68* | 0.71* | 1.00 | 0.23 | 0.75* | -0.46 | -0.26 |
| **Branch mass** | -0.04 | 0.16 | 0.27 | 0.49 | 0.29 | 0.35 | 0.22 | 0.23 | 1.00 | 0.08 | 0.09 | 0.38 |
| **Stem mass** | -0.30 | -0.17 | 0.51 | 0.65 | 0.39 | 0.52 | 0.46 | 0.75* | 0.08 | 1.00 | -0.13 | -0.12 |
| **H** | 0.16 | 0.31 | 0.24 | 0.30 | 0.15 | 0.09 | -0.27 | -0.46 | 0.09 | -0.13 | 1.00 | 0.45 |
| **D** | 0.07 | 0.18 | 0.31 | 0.21 | 0.17 | 0.14 | -0.44 | -0.26 | 0.38 | -0.12 | 0.45 | 1.00 |
